# Post-acute Sequelae of SARS-CoV-2 Infection and Subjective Memory Problems

**DOI:** 10.1001/jamanetworkopen.2021.19335

**Published:** 2021-07-01

**Authors:** Esther S. Oh, Tracy D. Vannorsdall, Ann M. Parker

**Affiliations:** Department of Medicine, Johns Hopkins University School of Medicine, Baltimore, Maryland; Department of Psychiatry and Behavioral Sciences, Johns Hopkins University School of Medicine, Baltimore, Maryland; Department of Pathology, Johns Hopkins University School of Medicine, Baltimore, Maryland; Johns Hopkins University School of Nursing, Baltimore, Maryland; Department of Psychiatry and Behavioral Sciences, Johns Hopkins University School of Medicine, Baltimore, Maryland; Department of Neurology, Johns Hopkins University School of Medicine, Baltimore, Maryland; Pulmonary and Critical Care Medicine, Johns Hopkins University School of Medicine, Baltimore, Maryland; Outcomes After Critical Illness and Surgery Research Group, Johns Hopkins University School of Medicine, Baltimore, Maryland

As of June 7, 2021, there are more than 168 000 000 survivors of COVID-19 worldwide. Approximately 72% of survivors report at least 1 symptom persisting 30 days or more beyond the acute illness, and symptoms frequently persist even among patients who were relatively young and never hospitalized.^1^ Commonly reported symptoms include fatigue, shortness of breath, anxiety, depression, insomnia, and cognitive impairment.^1^ Given the emerging public health crisis represented by this burden of survivorship, there is an urgent need for understanding postacute sequelae of SARS-CoV-2 (PASC).

Although much has been written about PASC, there is still little known about the long-term cognitive sequelae of COVID-19. The cohort study by Søraas et al^2^ is one of the largest cohort studies reporting on long-term cognitive symptoms of COVID-19. In this study, 13 001 individuals who were tested largely because they had symptoms suspicious for SARS-CoV-2 infection or individuals who were untested and randomly selected from the general population in Norway. The individuals who had been tested for SARS-CoV-2 were tested between February 1 and April 15, 2020, and none were hospitalized. Participants completed an online questionnaire including demographics, comorbidities, health-related quality of life, and memory problems at baseline and approximately 8 months later. After adjusting for confounders, SARS-CoV-2 test positivity was associated with an almost 5 times higher likelihood of reporting subjective memory problems at 8-month follow-up.

Current understanding of cognitive sequelae of COVID-19 is still largely limited to individuals who required hospitalization. Among hospitalized patients, objective deficits have been reported in verbal fluency, attention, working memory, processing speed, executive functioning, learning, and memory, with no clear pattern of cognitive weakness emerging across studies.^3,4^ Importantly, survivors of critical illness commonly experience cognitive impairment, with more than a third having scores on cognitive testing consistent with moderate traumatic brain injury at 1 year follow-up.^5^ Hence, understanding overlapping and distinct features of cognitive impairment following critical illness, and COVID-19 is a key research priority.

In recent years, there has been mounting evidence that subjective memory concerns are associated with increased risk of future cognitive impairment. In long-term studies with more than 4 years of follow-up, older adults with subjective memory concerns have increased risk of progression to mild cognitive impairment (27%) or dementia (14%).^6^ However, while subjective cognitive concerns may portend cognitive decline in some older individuals, it remains too early to determine if this is the case following COVID-19. At present, limited data from COVID-19 survivors suggest subjective concerns are not associated with concurrent objective cognitive deficits.^3,4,7^

Poor cognitive performance may be associated with severity of mental and physical impairment. A 2021 observational study^8^ of 1077 adults discharged with diagnosis of COVID-19 in UK identified 4 different recovery phenotypes (mild, moderate, severe, and very severe) using cluster analysis of mental, physical, and cognitive health based on patient reported outcomes and tests of physical performance at the 5-month follow up. Mental health (eg, depression, anxiety, etc) and physical health outcomes were associated with each other within each cluster, while cognitive function, measured by Montreal Cognitive Assessment, remained independent. Importantly, the “moderate” cluster, which had the most pronounced cognitive impairment, also had the highest mean age among the 4 clusters.^8^ Therefore, examination of subjective memory concerns and more in-depth evaluation of objective cognitive outcomes in the context of mental and physical health outcomes will be essential in determining the nature and trajectory of cognitive impairment following COVID-19. In addition, the trajectory of individuals with subjective memory concerns may differ by age, and a closer cognitive follow-up may be warranted in older individuals.

Limitations of the study by Søraas et al^2^ include the low overall response rate (24%) and potential bias in both participation and responses to survey questions introduced by knowing their SARS-CoV-2 test results. Additionally, there are no data on race or ethnicity, and the results are from a single country in Scandinavia.

To advance the current knowledge of cognitive impairment among COVID-19 survivors, longer prospective cohort studies that include individuals from different races and ethnicities are needed to determine symptom persistence, and more importantly, the potential association between subjective cognitive impairment and rate of objective cognitive impairment compared with age-, sex-, and education-adjusted norms. Moreover, the functional consequences of persistent cognitive dysfunction among individuals who may have been at the height of their work productivity before SARS-CoV-2 infection is an important area in need of further investigation. Finally, ongoing efforts to understand the underlying biological mechanism of the effect of SARS-CoV-2 on the brain will be important in identifying those at high risk of developing subsequent cognitive impairment.
